# Increased expression of the RI**α** subunit of the cAMP-dependent protein kinase A is associated with advanced stage ovarian cancer

**DOI:** 10.1038/sj.bjc.6690149

**Published:** 1999-02

**Authors:** H M McDaid, M T Cairns, R J Atkinson, S McAleer, D P Harkin, P Gilmore, P G Johnston

**Affiliations:** Department of Oncology, The Queen's University of Belfast, University Floor, Lisburn Road, Belfast, BT9 7AB, UK

**Keywords:** type I-cAMP-dependent protein kinase, reverse transcription polymerase chain reaction, ovarian cancer, cAMP

## Abstract

The primary element in the cAMP signal transduction pathway is the cAMP-dependent protein kinase (PKA). Expression of the RIα subunit of type I PKA is elevated in a variety of human tumours and cancer cell lines. The purpose of this study was to assess the prognostic importance of RIα expression in patients with ovarian cancer. We have evaluated the expression of RIα in a panel of human ovarian tumours (*n*= 40) and five human ovarian cancer cell lines using quantitative reverse transcription polymerase chain reaction (RT-PCR) and Western blot analysis. The human ovarian cell lines OAW42 and OTN14 express high endogenous levels of RIα mRNA and protein (at significantly higher mRNA levels than high tissue expressors, *P*< 0.05). The ovarian cell line A2780 expresses low endogenous levels of RIα mRNA and protein (also at higher mRNA levels than low tissue expressors, *P*< 0.05). Quantitative RT-PCR revealed no significant difference in RIα mRNA expression between different ovarian histological subtypes in this study. No associations were found between RIα mRNA expression and differentiation state. RIα mRNA expression was significantly associated with tumour stage (*P*= 0.0036), and this remained significant in univariate analysis (*P*= 0.0002). A trend emerged between RIα mRNA expression levels and overall survival in univariate analysis (*P*= 0.051), however, by multivariate analysis, stage remained the major determinant of overall survival (*P*= 0.0001). This study indicates that in ovarian epithelial tumours high RIα mRNA expression is associated with advanced stage disease. RIα expression may be of predictive value in ovarian cancer and may be associated with dysfunctional signalling pathways in this cancer type. *1999 Cancer Research Campaign*


*British Journal of Cancer* (1999) **79**(5/6), 933–939
© 1999 Cancer Research Campaign
Article no. bjoc.1998.0149

### Increased expression of the RIα subunit of the cAMP-dependent protein kinase A is associated with advanced stage ovarian cancer

**HM McDaid, MT Cairns, RJ Atkinson, S McAleer, DP Harkin, P Gilmore and PG Johnston**

Department of Oncology, The Queen's University of Belfast, University Floor, Belfast City Hospital, Lisburn Road, Belfast BT9 7AB, UK

**Summary** The primary element in the cAMP signal transduction pathway is the cAMP-dependent protein kinase (PKA). Expression of the RIα subunit of type I PKA is elevated in a variety of human tumours and cancer cell lines. The purpose of this study was to assess the prognostic importance of RIα expression in patients with ovarian cancer. We have evaluated the expression of RIα in a panel of human ovarian tumours ($n = 40$) and five human ovarian cancer cell lines using quantitative reverse transcription polymerase chain reaction (RT-PCR) and Western blot analysis. The human ovarian cell lines OAW42 and OTN14 express high endogenous levels of RIα mRNA and protein (at significantly higher mRNA levels than high tissue expressors, $P < 0.05$). The ovarian cell line A2780 expresses low endogenous levels of RIα mRNA and protein (also at higher mRNA levels than low tissue expressors, $P < 0.05$). Quantitative RT-PCR revealed no significant difference in RIα mRNA expression between different ovarian histological subtypes in this study. No associations were found between RIα mRNA expression and differentiation state. RIα mRNA expression was significantly associated with tumour stage ($P = 0.0036$), and this remained significant in univariate analysis ($P = 0.0002$). A trend emerged between RIα mRNA expression levels and overall survival in univariate analysis ($P = 0.051$), however, by multivariate analysis, stage remained the major determinant of overall survival ($P = 0.0001$). This study indicates that in ovarian epithelial tumours high RIα mRNA expression is associated with advanced stage disease. RIα expression may be of predictive value in ovarian cancer and may be associated with dysfunctional signalling pathways in this cancer type.

**Keywords:** type I-cAMP-dependent protein kinase; reverse transcription polymerase chain reaction; ovarian cancer; cAMP

Ovarian cancer is the leading cause of death from gynaecological malignancies in the US (Parker et al, 1996) and occurs with highest frequency in Northern and Western Europe and North America (Whelan et al, 1992). Five-year survival rates for patients with stage III disease are approximately 15–20%, and less than 5% for patients with stage IV disease (NIH Consensus Statement, 1994). The diagnosis of ovarian disease at an advanced tumour stage hinders the prospects of survival and cure. The majority of patients respond well to platinum-based therapies, but most subsequently relapse and develop progressive disease. Although early diagnosis is a primary goal, secondary aims are to identify those patients with borderline and early stage tumours who will rapidly progress and develop metastatic disease. A marker of such prognostic value could identify those patients who may benefit from aggressive therapy.

The primary mediator of cAMP events in eukaryotes is the cAMP-dependent protein kinase (PKA) (Walsh et al, 1968), which is composed of an inactive tetramer containing two regulatory (R) subunits associated as a dimer and two catalytic subunits (C). There are two types of PKA, type I and type II, which contain the same C subunit but differ in the R subunit they contain (RI and RII respectively) (Reimann et al, 1971). Evidence suggests that overexpression of the type I PKA is associated with proliferation and cell transformation (Cho-Chung, 1990), therefore type I RI isoforms are thought to induce cell growth. Conversely, low expression of type I PKA (with or without alterations in the level of RII isoforms) correlates with terminal differentiation and growth inhibition (Cho-Chung, 1993). The mammalian ovary is under constant stimulation by pituitary gonadotropins, luteinizing hormone (LH) and follicle-stimulating hormone (FSH), which modulate the expression of numerous gonadal genes and exert their effects by activating cAMP synthesis through adenylate cyclase (Land, 1987). In view of the role of type I PKA in the regulation of cellular growth and differentiation, we have speculated that dysregulation of the PKA pathway may have profound effects on ovarian pathology. In this study, we have evaluated the expression of the RIα subunit of the type I cAMP-dependent protein kinase (PKA) in patients with ovarian cancer and in human ovarian cancer cell lines.

*Received 30 December 1997*
*Revised 22 May 1998*
*Accepted 2 June 1998*

*Correspondence to:* PG Johnston

### MATERIALS AND METHODS

### Cell culture

All ovarian cell lines were derived from serous cystadenocarcinomas of the ovary, except for the OTN14 cell line which was derived from a mucinous cystadenocarcinoma. The PEA1 and PEA2 ovarian cell lines were kindly provided by Dr S Langdon (Langdon et al, 1988). OTN14 was kindly provided by Dr L Poels (Van Niekerk et al, 1988), OAW42 cells from Dr A Wilson (Wilson, 1984) and A2780 cells from Dr RI Freshney. All cell lines were maintained in RPMI (GibcoBRL) supplemented with 10% heat-inactivated fetal calf serum (Imperial), penicillin–streptomycin (100 mg $ml^{-1}$), sodium pyruvate (100 mg $ml^{-1}$) and insulin (10 mg $ml^{-1}$) in a humidified 5% carbon dioxide atmosphere.

### Tumour collection

Tissue samples from patients with suspected ovarian epithelial carcinoma were collected at initial debulking surgery. Samples were stored in liquid nitrogen upon collection and transferred to vials for long-term storage at –70°C, or in vials suspended in liquid nitrogen. Clinicopathological details, such as histology, grade and differentiation state were determined by an ovarian pathologist and retained on a computer database for future reference.

### RNA extraction

Cells ($10^7$) were lysed by passing through a fine needle and syringe in 0.5–1 ml of Total RNA Isolation Reagent (Advanced Biotechnologies), followed by chloroform extraction and precipitation according to the manufacturer's instructions. The isolation procedure extracted DNA and proteins into an organic phase and interphase which was discarded, eliminating the need for DNAase treatment before polymerase chain reaction (PCR). Negative PCR controls containing RNA instead of cDNA confirmed the absence of contaminating genomic DNA. Approximately 100 mg of tissue samples were homogenized (Ultra-turrax T8, IKA laboratories, Germany) in 1 ml of isolation reagent at 4°C. Pelleted RNA was resuspended in DNAase-RNAase-free distilled water (Sigma) and quantified by measuring absorbance at 260 and 280 nm (LKB Ultraspec II). Samples were also electrophoresed on 1% agarose gels containing ethidium bromide (10 mg $ml^{-1}$) to verify sample integrity and the exclusion of genomic DNA. RNA was stored at –70°C.

### Quantitative reverse transcription polymerase chain reaction (RT-PCR)

Total RNA (5 μg) was reverse transcribed in a 50-μl reaction volume containing 300 U of MMLV reverse transcriptase (GibcoBRL) in the presence of oligo(dT) at a final concentration of 0.02 μg $\mu l^{-1}$ (Pharmacia Biotech), 60 U RNAguard (Pharmacia Biotech), 0.5 mM dNTPs, 10 mM DTT and 1 × RT buffer at 37°C for 1 h, followed by 95°C for 5 min. All reactions used DNAase-/RNAase-free distilled water and cDNAs were stored at –70°C. The gene for the S14 ribosomal protein was used as an internal standard for quantification of RIα mRNA (Leonard et al, 1993) (accession number M13934). S14 primers amplify a fragment of 143 bp from cDNA. S14HumS sense strand (nucleotides 187–208): 5′-GGC AGA CCG AGA TGA ATC ATA A-3′. S14HumA antisense strand (nucleotides 331–311):5′-CAG GTC CAG GGG TCT TGG TCC-3′.

RIα primers were designed as areas of minimal homology with other R isoforms and yield an amplicon of 338 bp with cDNA. This fragment codes entirely for mRNA, which is translated into protein (accession no M33336).

RIαP1 sense strand (nucleotides 648–667): 5′-AAA GAA TGG GCA ACC AGT G-3′. RIαP2 antisense strand (nucleotides 986–976): 5′-GCT GAC CCC TCT AAA ATA ATG-3′. All primer sets were manufactured by R & D systems, UK. Expression of RIα mRNA was described as a ratio of S14 expression levels.

cDNA (2 μl) was amplified in an 18-μl PCR mix containing 0.25 U *Taq* DNA polymerase, 1.5 mM magnesium chloride and 1 × *Taq* buffer (all Thermoprime Plus, Advanced Biotechnologies), 1 μM S14HumA, S14HumS, RIαP1 and RIαP2, and 2.5 μCi [$^{32}P$]-dCTP (3000 Ci $mmol^{-1}$). Thermocycling was carried out on a Perkin Elmer Cetus 480: after an initial melt at 94°C for 4 min, samples were amplified through 18 cycles of 94°C for 1 min, 54°C for 2 min and 72°C for 30 s, with a final incubation at 72°C for 5 min. The linear range of amplification was established at 18 cycles. Samples were amplified in duplicate and PCR products were denatured at 95°C and electrophoresed on 6% polyacrylamide gels (Ultragel Sequagel Complete, National Diagnostics). Gels were dried at 80°C for 40 min (Rapidry-electrophoresis ATTO, Japan) and exposed to radiographic film in a cassette with intensifying screens at –70°C for a period of 24–96 h. Radiolabelled products were analysed using a densitometer (Bio-rad GS670, Molecular Analyst software).

### Optimization of RT-PCR

The series of cell lines described earlier were used to optimize the assay conditions. PCR standardization was found to be an important factor in obtaining reliable results, and extensive validations of the technique with regard to reproducibility and PCR amplification efficiency were carried out. Cycle optimization determined the linear range of amplification to be 18 cycles (assuming a standard cDNA concentration of 5 μg and taking into account the variability in RIα mRNA expression in the cell lines). Reproducibility was achieved by preparing all reactions from a 'master mix' of appropriate reagents. Negative controls were routinely included by amplifying blanks containing distilled water, and genomic DNA, to verify primer specificity and amplicon size. Exposure times of dried gels were monitored and analysis ratios for which S14 values exceeded 6.0 were excluded in the calculation of the overall value.

### Western blot analysis

Cell pellets were sonicated in 50–200 μl of 0.1 M KPhos lysis buffer, pH 7.4 (1 M potassium dihydrogen phosphate:1 M dipotassium hydrogen phosphate; 4:1) in the presence of protease inhibitors (0.1 mM phenylmethylsulphonyl fluoride, 3.3 μg $\mu l^{-1}$ aprotinin and 10 μg $\mu l^{-1}$ leupeptin). Lysates were centrifuged at 14 000 r.p.m. for 5 min at 4°C to remove cell debris and the supernatent recovered to fresh tubes. Total protein was quantified against a bovine serum albumin standard using the method of Bradford (1976). Equal quantities of protein (10–100 μg) were resolved on 7.5–10% polyacrylamide gels using the Mini-PROTEAN II electrophoresis cell and transfer system (Bio-rad). Ten microlitres of prestained sodium dodecyl sulphate polyacrylamide gel electrophoresis (SDS-PAGE) high-range standards (Bio-rad) was also loaded onto gels to determine optimal electrophoretic separation of proteins. Proteins were transferred for 2 h in transfer buffer (48 mM Tris, 39 mM glycine and 0.5 M EDTA in 20% methanol). Nitrocellulose blots were incubated for 30 min at room temperature in blocking solution [phosphate-buffered saline (PBS)/0.1% Tween/5% blocking agent]. Filters were washed three times in PBS/0.1% Tween, and incubated with a 1:1000 dilution of anti-protein kinase A RIα monoclonal antibody (Transduction Laboratories, UK) in PBS/0.1% Tween/5% block overnight at 4°C (or at room temperature for 1 h). Blots were washed three times in PBS/0.1% Tween and secondary antibody applied [anti-mouse horseradish peroxidase conjugated (Amersham, UK)] at a 1:2000 dilution at room temperature for 1 h in PBS/0.1% Tween/5% block. Blots were washed three times in PBS/0.1% Tween. The chemiluminescent substrate based on the enhanced chemiluminescence (ECL) method of Amersham was applied to blots for 1 min.

© Cancer Research Campaign 1999

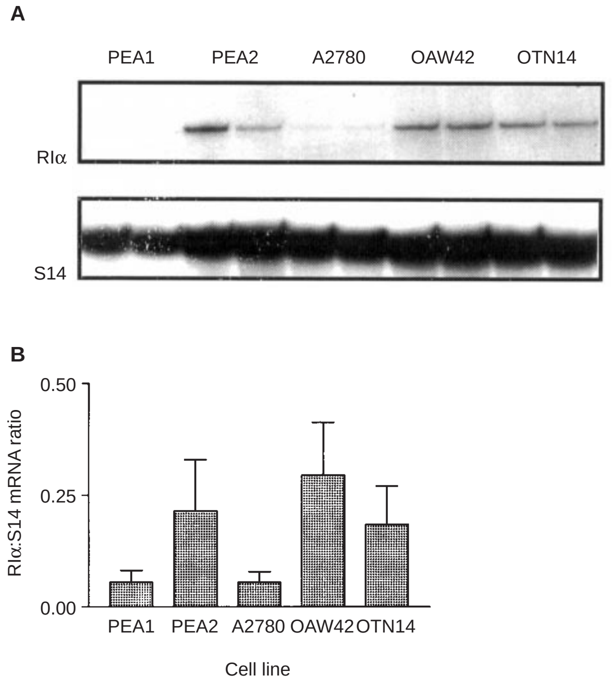

**Figure 1** (**A**) Autoradiograph showing RIα and S14 mRNA expression levels in the five cell lines. Samples were amplified in duplicate. (**B**) Composite graph depicting the average distribution of RIα mRNA levels in five ovarian cancer cell lines (± s.e.)

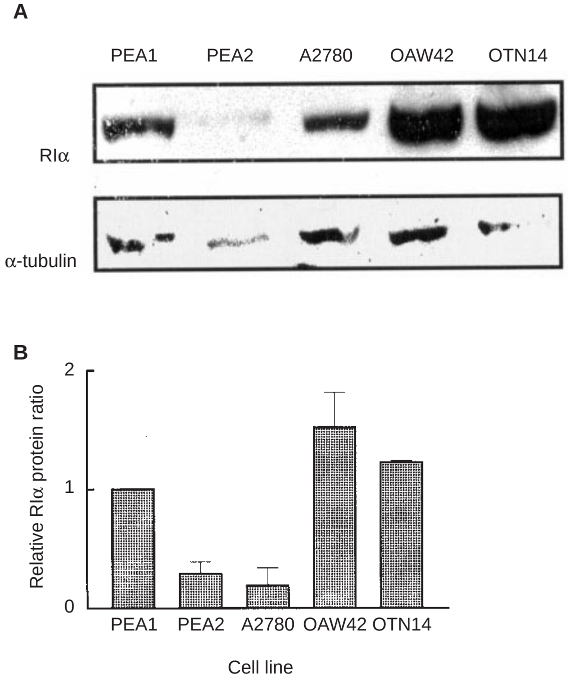

**Figure 2** (**A**) Autoradiograph depicting RIα and α-tubulin protein expression in the five cell lines. (**B**) Composite graph depicting the average distribution of RIα protein in the cell lines (± s.e.). Each protein value is standardized against the value obtained for PEA1, which is assigned an arbitrary value of 1.0

Excess reagent was drained and blots wrapped in cling film and exposed to radiographic film for periods of between 10 s and 1 h. The size of detected proteins was estimated by comparison with the location of prestained standards on the blot. Quantitative analysis was carried out by densitometry. Blots were subsequently stripped by immersing in buffer (1 M glycine hydrochloride, pH 2.8, 4 M sodium chloride) at room temperature for 1 h followed by three washes with copious volumes of PBS/0.1% Tween, before reprobing with an anti-α-tubulin mouse monoclonal antibody (Sigma, UK) at 1:1000 dilution in PBS/0.1% Tween/2.5% block. Expression of RIα protein was described as a ratio of α-tubulin expression levels, and the cell line PEA1 was assigned a relative value of 1.0.

### Statistical analysis

The mean RIα:S14 ratios and the relationships between variables were analysed using non-parametric statistical tests (Mann–Whitney *U*-test, Kruskal–Wallis) using the software SPSS. Cox regression models were used to evaluate the relationship between RIα mRNA expression and clinical features of each tumour. These models were used to test whether overall survival was independent of prognostic factors (stage, grade and histology). All *P*-values are two-sided. Overall survival times were calculated from the date of first surgery to death.

### RESULTS

### Expression of RIα mRNA and protein in six human cancer cell lines

Quantifiable levels of RIα mRNA were detected in all five cell lines (mean RIα mRNA expression = 0.161 ± 0.104) with a 4.8-fold difference in RIα mRNA expression ratios between the highest and lowest expressors, OAW42 and A2780 respectively (Figure 1). RIα mRNA expression was highest in the cell line OAW42 (mean RIα mRNA expression = 0.295) and lowest in the cell lines A2780 and PEA1 (mean RIα mRNA expression = 0.055). The cell lines PEA2 and OTN14 also expressed high levels of RIα mRNA. Mann–Whitney *U*-test analysis of the data confirms a significant difference in the RIα mRNA expression levels of OAW42 cells (high expressors) compared with A2780 and PEA1 (low expressors) ($P < 0.05$, $n = 5$).

RIα protein levels were determined using a monoclonal antibody (Figure 2) (mean RIα protein expression = 0.847 ± 0.584). The cell lines A2780 and PEA2 expressed the lowest endogenous levels of RIα protein, whereas OAW42 cells expressed the highest levels. RIα protein expression was also relatively high for the cell lines PEA1 and OTN14. The levels of RIα mRNA and protein expression are consistent with respect to the cell lines A2780, OAW42 and OTN14, however there is an inverse relationship between RIα mRNA and protein expression in the cell lines PEA1 and PEA2 and, therefore, a poor statistical correlation in a comparison of all cell lines ($r^2 = 0.266$; $P > 0.5$). In a comparison of cell line data, excluding PEA1 and PEA2, there is a good statistical correlation between RIα mRNA and protein expression levels ($r^2 = 0.932$). Moreover, in a preliminary investigation of RIα protein expression levels in the tumours examined, there was also a good correlation between RIα mRNA and protein expression levels ($r^2 = 0.86$).

### Expression of RIα mRNA in human ovarian epithelial tissue

Quantifiable levels of RIα mRNA were detected in all tumours: serous, $n = 15$; mucinous, $n = 14$; endometroid, $n = 7$; clear cell,

© Cancer Research Campaign 1999

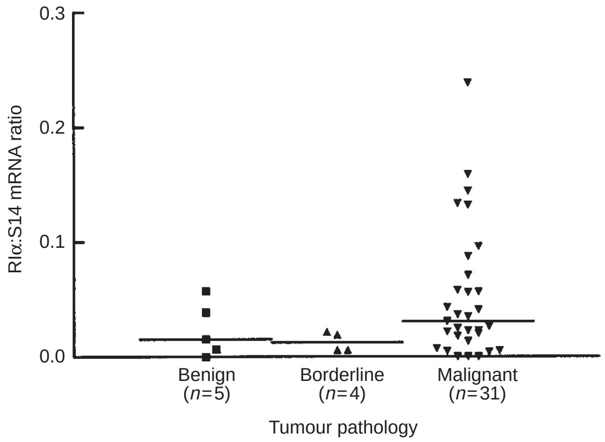

**Figure 3** RIα mRNA expression levels in malignant, borderline and benign ovarian tumours. No significant differences were observed between tumour groups. Horizontal bars, median values

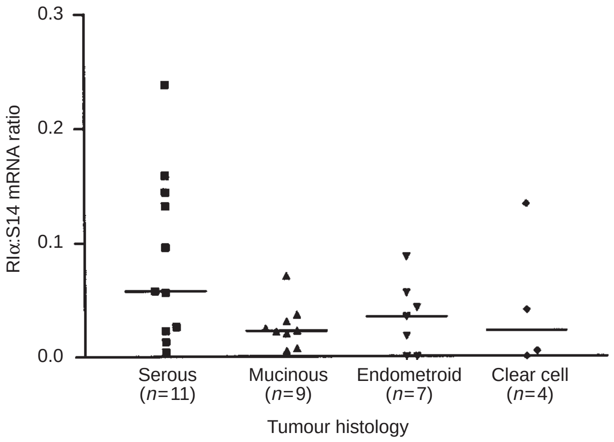

**Figure 4** RIα mRNA expression levels in histological subtypes of malignant ovarian tumours. No significant differences were observed between tumour histologies. Horizontal bars, median values

**Table 1** Association of RIα mRNA expression with clinicopathological features

| Variable | Classification | *P*-value |
|---|---|---|
| Pathology | Benign<br>Borderline<br>Malignant | 0.0871* |
| Histology | Serous<br>Mucinous<br>Endometrioid<br>Clear cell | 0.201* |
| Histology | Serous<br>Mucinous | 0.0683** |
| Stage | Benign<br>Borderline<br>Stage I/II<br>Stage III/IV | 0.0036* |
| Stage | Stage I/II<br>Stage III/IV | 0.001** |
| Differentiation state | Well/moderate<br>Poor | 0.568* |

*Kruskal–Wallis test; **Mann–Whitney *U*-test.

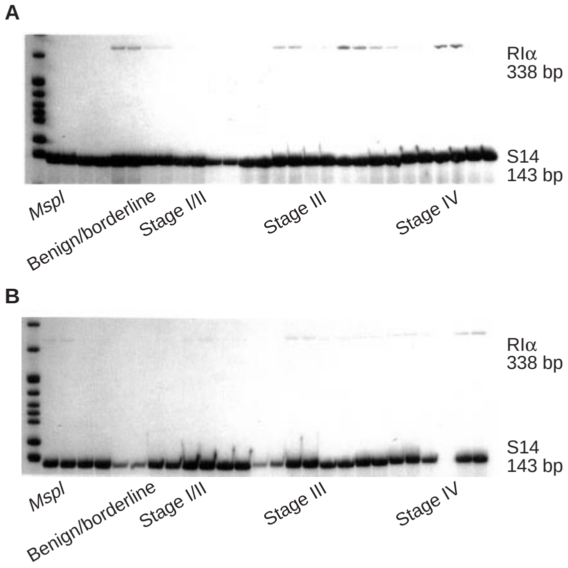

**Figure 5** Levels of RIα and control S14 mRNA expression in (**A**) serous and (**B**) mucinous ovarian tumours after RT-PCR for 18 cycles as described in Materials and methods

*n* = 4. RIα mRNA was detectable at low levels in one benign serous tumour. Unfortunately, the number of benign and borderline ovarian tumours were underrepresented in this study because of poor availability. Expression ratios were found to vary extensively among samples with a 1192-fold difference in RIα expression between the highest and lowest expressors (ratios ranging from 0.0002 to 0.2384), and data was skewed towards the lower levels. RIα mRNA expression levels were divided into two malignant groups for the analysis of survival data, RIα high (*n* = 15) compared with RIα low (*n* = 16), based on expression levels below and above the median RIα mRNA expression level.

### Histopathological correlations

There was a trend for higher RIα mRNA expression levels in malignant ovarian tumours than benign and borderline tumours, however, this was not significant (*P* = 0.0871) (Figure 3; Table 1). The pooled median RIα mRNA expression levels for serous tumours was 0.058 ± 0.0224, for mucinous tumours 0.0227 ± 0.006, for endometrioid tumours 0.035 ± 0.012 and for clear cell tumours 0.023 ± 0.031. There was also a trend for serous tumours to express higher levels of RIα mRNA than mucinous tumours, however this difference was not significant (*P* = 0.068) nor were there significant statistical differences in RIα mRNA expression of all histological subtypes (*P* = 0.201) (Figure 4). Figure 5A and B depicts the RT-PCR autoradiographs obtained from serous and mucinous tumour histologies respectively.

No significant difference was detected between RIα mRNA expression levels of well/moderately and poorly differentiated tumours of all histological subgroups (*P* = 0.568), nor was any association observed in a subset analysis of serous and mucinous

 © Cancer Research Campaign 1999

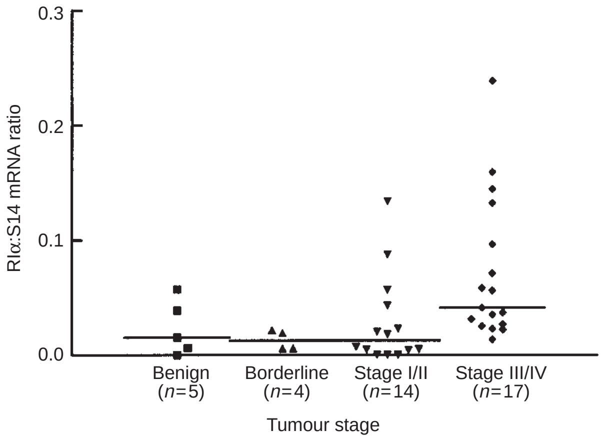

**Figure 6** RIα mRNA expression levels measured relative to S14 mRNA expression in benign, borderline, stage I, II, III and IV ovarian tumours. There is a significant difference between the medians of each subgroup (*P* = 0.0036), and between the medians of stage I/II and stage III/IV tumours (*P* = 0.001). Horizontal bars, median values

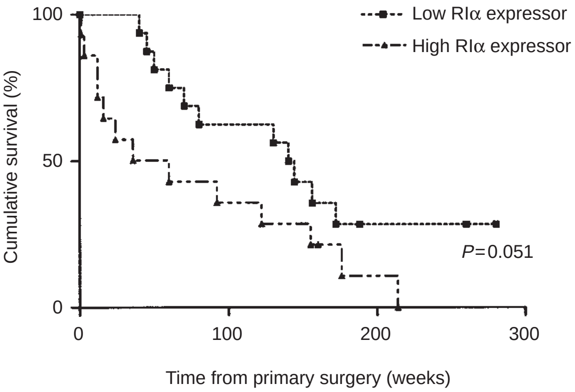

**Figure 7** Association of RIα mRNA expression with overall survival in patients with ovarian cancer. This relationship has a trend towards statistical significance, with patients expressing higher levels of RIα mRNA exhibiting shorter overall survival rates. Low RIα expressor: number of cases = 16, number of events = 11; high RIα expressor: number of cases = 15, number of events = 13

**Table 2** Significance levels from Cox models to test for the association of RIα mRNA expression with overall survival controlling for other prognostic factors

| Variable | Classification | RIα low (*n*) | RIα high (*n*) | Univariate *P*-value* |
|---|---|---|---|---|
| Histology | Serous | 3 | 8 | 0.737 |
| | Mucinous | 5 | 4 | |
| | Endometrioid | 3 | 4 | |
| | Clear cell | 2 | 2 | |
| Histology | Serous | 3 | 8 | 0.0205 |
| | Mucinous | 5 | 4 | |
| Stage | Benign | 3 | 2 | 0.0002 |
| | Borderline | 4 | 0 | |
| | Stage I/II | 10 | 4 | |
| | Stage III/IV | 3 | 14 | |
| Differentiation state | Well/moderate | 7 | 6 | 0.0375 |
| | Poor | 6 | 10 | |
| RIα status | Low | *n* = 16 | – | 0.051 |
| | High | *n* = 15 | – | |

*Log-rank statistic based on Cox models. RIα mRNA expression divided into two categories, RIα high/RIα low, which correspond to values below and above the median value.

tumour grades. Tumour grade was not associated with RIα mRNA expression in ovarian tumours. Univariate analysis of grade and histological subtype (for serous and mucinous only) indicated significant associations with overall survival (*P* = 0.0375 and *P* = 0.0205 respectively).

### Relationship to tumour stage

RIα mRNA expression levels were highest in stage III and IV tumours (*n* = 17; median RIα mRNA expression = 0.041 ± 0.0628), regardless of histology, and lowest in benign, borderline and stage I and II ovarian tumours (*n* = 23; median RIα mRNA expression = 0.0151 ± 0.0322). These differences were true for all histological types (*P* = 0.0036; Figure 6). This significance increases in a comparison of stage I/II with stage III/IV tumours (*P* = 0.001). Therefore, regardless of histology, increased RIα mRNA expression was associated with advanced stage ovarian tumours. The association of RIα mRNA expression with tumour stage remains significant in an analysis of serous and mucinous histologies individually (*P* < 0.01).

### Clinical associations

The median survival time for RIα low mRNA expressors was 142 weeks compared with 60 weeks for the RIα high subgroup (Figure 7). Log-rank analysis of the data indicated a trend between RIα mRNA expression levels and overall survival by univariant analysis (*P* = 0.051), however this is lost by multivariate analysis (Table 2).

Cox models were used to evaluate the association of RIα mRNA expression with overall survival, controlling for the clinical parameters of histology, stage and grade (Table 2). Tumour stage had the most significant statistical association with overall survival (*P* = 0.0002, univariate analysis). Moreover, by multivariate analysis, stage remained significant (*P* = 0.0001–0.0002),

© Cancer Research Campaign 1999 

after adjusting for RIα mRNA status, histology and grade. Thus, although RIα expression is associated with stage and survival, tumour stage was the most significant predictor of survival in this cohort.

### DISCUSSION

In this study, we have examined the expression of RIα in patients with ovarian cancer. The results indicate that elevated levels of RIα mRNA show a very significant correlation with advanced stage ovarian cancer ($P$ = 0.0036). There was no statistically significant relationship between RIα mRNA expression and patient survival. However, there was a trend towards this, in which those patients whose tumours expressed lower levels of RIα mRNA expression had longer overall survival rates ($P$ = 0.051).

In contrast to this study, an analysis of PKA binding proteins from 107 human ovarian tumours suggests that both total cAMP-binding protein levels and photoaffinity-labelled RI protein levels were not significantly associated with stage ($P > 0.5$) (Simpson et al, 1996). Given the poor correlation between RIα mRNA and protein found with our panel of cell lines, this may provide a possible explanation for the different findings of both studies. The findings presented here underline the potential role of the RIα subunit overexpression with respect to advanced tumour stage and subsequent poor prognosis. The accurate determination of the prognostic significance of RIα mRNA expression will require a prospective study utilizing large-sample analysis.

RIα overexpression may also be useful as a tool for monitoring disease progression and response to treatment. Unfortunately, there was only a small number of matched pairs available in our ovarian tumour bank which did not reveal a significant trend. A more extensive study of RIα expression in breast cancer patients after treatment with tamoxifen has been reported (Miller et al, 1996). From the 37 matched tumour pairs available, decreases in RIα expression levels were observed in 18 patients, increases in ten patients, and no change in RIα status in nine patients. Seventeen of the 18 patients with decreased levels of RIα expression showed tumour regression, indicating that RIα expression may have potential as a prognosticator of clinical response. Current therapeutic strategies which target PKA include the cAMP analogue 8-Cl-cAMP, which is currently in phase I/II clinical trial (Tortora et al, 1995), and RIα antisense oligonucleotides (Nesterova et al, 1995). Both approaches involve the modulation of endogenous PKA I/II intracellular ratios via the down-regulation of RI isoforms. Because endogenous expression levels of RIα determine the concentration of 8-Cl-cAMP required to induce growth inhibition (McDaid et al, 1997), the future therapeutic efficacy of both may rely on the determination of endogenous RIα expression levels in patients who are most likely to benefit from PKA-targeted treatment.

In this study, RIα mRNA expression was not associated with tumour pathology or grade and we did not find statistically significant differences between the RIα mRNA expression levels of serous compared with mucinous, endometrioid or clear cell tumours, although a trend was apparent for serous tumours to express higher levels of RIα mRNA. Simpson et al (1996) have found an association between elevated RI protein and the serous histological subgroup ($P$ = 0.01). The consequence of elevated levels of RIα expression for serous tumours would be a relative up-regulation of PKAI activity compared with PKAII activity, but, overall, a decrease in free catalytic subunits and a subsequent reduction in phosphorylation of proteins by this pathway. Because it is believed that the cAMP pathway generally antagonizes the growth factor-stimulated pathway (Beebe, 1994), this decrease in downstream signalling by the cAMP pathway may remove this inhibition and so promote cell proliferation in an already dysfunctional cell.

This study has also addressed the expression of RIα mRNA in a panel of ovarian cell lines. Endogenous RIα in these ovarian cell lines can be divided into two categories, low expressors (PEA1 and A2780), which express at levels comparable to those found in tissue samples expressing high levels, and high expressors (PEA2, OAW42 and OTN14), whose expression greatly exceeds levels determined in all tissue samples ($P < 0.05$). One simple observation for the difference in expression levels between the two groups is a difference in the DNA content of the cells. The two low expressors, PEA1 and A2780, have pseudodiploid modal chromosome numbers of between 41 and 50 whereas those cell lines which displayed greatly elevated levels of RIα have modal chromosome numbers of ≥ 80, therefore gene amplification may explain this observation.

In conclusion, the level of RIα mRNA expression is associated with advanced stage ovarian cancer, but not with overall survival, although there is a trend by univariate analysis between high levels of RIα mRNA expression and poor patient survival. A prospective study exploiting larger sample numbers will be necessary to fully evaluate the role of RIα as a prognosticator of survival.

### ACKNOWLEDGEMENTS

This work was funded by grants from the Ulster Cancer Foundation and the Department of Education for N-Ireland (DENI).

### REFERENCES

Beebe SJ (1994) The cAMP-dependent protein kinases and cAMP signal transduction. *Semin Cancer Biol* **5**: 285–294

Bradford MM (1976) A rapid and sensitive method for the quantification of microgram quantities of protein utilising the principle of protein-dye binding. *Anal Biochem* **72**: 248–254

Cho-Chung YS (1990) Role of cAMP receptor proteins in growth, differentiation, and suppression of malignancy: new approaches to therapy. *Cancer Res* **50**: 7093–7100

Cho-Chung YS (1993) Differentiation therapy of cancer targeting the RIα regulatory subunit of cAMP-dependent protein kinase. *Int J Oncol* **3**: 141–148

Land JA (1987) Ovulation, ovulation induction and ovarian carcinoma. *Baillières Clin Obstet Gynaecol* **43**: 455–470

Langdon SP, Lawrie SS and Hay FG (1988) Characterisation and properties of nine human ovarian adenocarcinoma cell lines. *Cancer Res* **48**: 6166–6172

Leonard MW, Lim K-C and Engel JD (1993) Expression of the chicken GATA factor family during early erythoid development and differentiation. *Development* **119**: 519–531

McDaid H, Cairns MT, Church S, White P and Johnston PG (1997) Response to 8-Cl-cAMP in a panel of human cancer cell lines is dependent on intrinsic RIα expression levels. *Proc Am Assoc Cancer Res* **38**: abstract 2823

Miller WR, Hulme MJ, McCallum J, Cameron DA, Dixon JM and Bartlett JMS (1996) Tumour RIα mRNA and response to tamoxifen in patients with breast cancer. *Proc Am Assoc Cancer Res* **37**: abstact 1627

Nesterova M and Cho-Chung YS (1995) A single-injection protein kinase A-directed antisense treatment to inhibit tumour growth. *Nature Med* **1**: 528–533

NIH Consensus Statement (1994) Ovarian cancer: screening, treatment and follow-up. In *NIH Consens Statment* **12**: 1–30

Parker SL, Tong T, Bolden S and Wingo PA (1996) Cancer statistics, 1996. *CA Cancer Clin* **46**: 5–27

Reimann KV, Walsh DA and Krebs EG (1971) Purification and properties of rabbit

 © Cancer Research Campaign 1999

skeletal muscle adenosine 3′,5′-monophosphate-dependent protein kinases. *J Biol Chem* **246**: 1986

Simpson BJB, Ramage AD, Hulme MJ, Burns DJ, Katsaros D, Langdon SP and Miller WR (1996) Cyclic adenosine 3′,5′-monophosphate-binding proteins in human ovarian cancer: correlations with clinicopathological features. *Clin Cancer Res* **2**: 201–206

Tortora G, Ciardiello F, Pepe S, Tagliaferri P, Ruggiero A, Bianco C, Guarrasi R, Miki K and Raffaele Bianco A (1995) Phase I clinical study with 8-Cl-cAMP and evaluation of immunological effects in cancer patients. *Clin Cancer Res* **51**: 1600–1605

Van Niekerk CC, Poels LG, Jap PH, Smeets DF, Thomas CM, Ramaekers FC and Voous GP (1988) Characterisation of a human ovarian carcinoma cell line, OTN14 derived from a mucinous cystadenocarcinoma. *Int J Cancer* **42**: 104–111

Walsh DA, Perkins JP and Krebs EG (1968) An adenosine 3′,5′-monophosphate-dependent protein kinase from rabbit skeletal muscle. *J Biol Chem* **243**: 3763–3765

Whelan SL and Ferlay J (1992) Cancer incidence in five continents. Age-specific and standardized incidence rates. *IARC Sci Publ* 120: 178–861

Wilson AP (1984) Characterisation of a cell line derived from the ascites of a patient with papillary serous cystadenoma of the ovary. *J Natl Cancer Inst* **72**: 513–521

© Cancer Research Campaign 1999
